# Gut Microbiome–Metabolite Interactions Contribute to Esophageal Cancer Risk: Evidence From Mendelian Randomization and Multiomics Integration

**DOI:** 10.1155/ijog/5967802

**Published:** 2026-06-08

**Authors:** He Yang, Yi Liu, Tianyu Zhu, Zhihan Xiao, Hongya Wang, Shusheng Zhu, Yongbing Chen

**Affiliations:** ^1^ Department of Thoracic Surgery, The Second Affiliated Hospital of Soochow University, Suzhou, China, suda.edu.cn; ^2^ Department of Digestive System, Anqing Municipal Hospital, Anqing, China, aqslyy.com.cn; ^3^ The First Clinical Medical College, Bengbu Medical University, Bengbu, China, bbmc.edu.cn; ^4^ Department of Thoracic Surgery, Tongji Hospital, Huazhong University of Science and Technology, Wuhan, China, hust.edu.cn; ^5^ Department of Thoracic Surgery, Taizhou Hospital of Traditional Chinese Medicine, Taizhou, China

**Keywords:** esophageal cancer, gut microbiota, metabolite, multiomics

## Abstract

**Background:**

Growing evidence implicates gut microbiota (GM) in the pathogenesis of esophageal cancer (EC). However, the causal nature of this association—particularly the potential mediating role of circulating metabolites—remains insufficiently clarified, especially across EC subtypes.

**Methods:**

We applied a two‐sample Mendelian randomization (MR) framework to investigate the causal associations of GM and blood metabolites with EC and esophageal adenocarcinoma (EAC). The primary analysis was conducted using the inverse‐variance weighted method, with complementary MR approaches and genetic risk score (GRS) validation to ensure robustness. Mediation analyses were further performed to assess whether metabolites mediate the effects of GM on EC and EAC.

**Results:**

Twenty‐five GM taxa showed significant associations with EC, including 11 with risk‐promoting and 14 with protective effects. For EAC, 15 taxa were implicated, with 11 increasing and 4 decreasing disease risk. In addition, five metabolites were causally linked to EC (two risk‐related and three protective), and six to EAC (five risk‐related and one protective). Mediation analyses revealed that specific metabolites partially mediated the effects of GM on both EC and EAC.

**Conclusions:**

Our findings provide genetic evidence for a causal GM–metabolite–cancer axis in EC pathogenesis. These results highlight the role of circulating metabolites as potential intermediaries linking GM to EC and EAC, and suggest new avenues for biomarker discovery and microbiome‐targeted interventions.

## 1. Introduction

Esophageal cancer (EC) remains a major global health burden, ranking ninth in incidence and sixth in cancer‐related mortality worldwide [1, 2]. EC primarily comprises two histological subtypes: esophageal squamous cell carcinoma (ESCC) and esophageal adenocarcinoma (EAC) [3]. ESCC accounts for approximately 80% of cases and can arise throughout the esophagus [1], whereas EAC—representing around 20% of cases—predominantly affects individuals in developed countries, particularly Caucasian populations, and typically occurs in the distal esophagus or at the gastroesophageal junction [4]. Notably, despite its lower prevalence, the incidence of EAC has surged by nearly 60% in recent decades, making it one of the fastest rising cancers [5].

Patients with EC are often diagnosed at an advanced stage and usually require neoadjuvant chemoradiotherapy or perioperative chemotherapy followed by surgical resection [6, 7]. However, disease recurrence remains common, even after comprehensive multimodal therapy, leading to poor long‐term survival [8, 9]. Established risk factors for EC include tobacco smoking, alcohol consumption, opium use, air pollution, and dietary patterns characterized by low fruit and vegetable intake and high consumption of red meat and pickled foods [10]. Nevertheless, the multifactorial nature of EC suggests that additional, as yet unidentified, contributors may underlie its pathogenesis. Among these, the gut microbiota (GM) has emerged as a promising yet underexplored factor [11].

The GM represents the largest and most diverse microbial ecosystem in the human body [12]. Mounting evidence links GM dysbiosis to a range of diseases, including cancer. In EC, alterations in the GM composition may influence carcinogenesis via various mechanisms. For instance, high‐fat or high‐fructose diets can reshape the gut microbial landscape, inducing metabolic disturbances, systemic inflammation, and immune dysregulation—conditions conducive to tumor initiation and progression [11]. Additionally, differences in fecal microbial profiles have been observed between EC patients and healthy individuals [13]. Although these associations are compelling, establishing a direct causal link remains challenging due to confounding variables.

Metabolites—comprising the substrates and by‐products of host and microbial metabolism—offer a dynamic readout of physiological and pathological states. Metabolomics, which enables the comprehensive profiling of metabolites in biological fluids, has become a powerful tool for disease biomarker discovery, particularly in oncology [14]. Numerous studies have demonstrated the diagnostic potential of metabolites in early cancer detection using plasma, serum, or urine samples [14–18]. However, the application of metabolites as reliable biomarkers is hindered by substantial interindividual variability, driven by both genetic and environmental influences [19]. The precise interplay between metabolic alterations and disease development, especially in EC, remains incompletely understood.

Given that both GM and metabolites are implicated in EC, we hypothesize that metabolites may mediate the effect of GM on disease risk. Mendelian randomization (MR), an analytical framework that leverages genetic variants as instrumental variables (IVs), provides a robust approach to infer causal relationships by minimizing confounding and reverse causality [20]. MR‐based mediation analysis further enables dissection of potential causal pathways. In this study, we applied outcome‐wide MR to systematically investigate the causal relationships among GM, metabolites, and EC, aimed at elucidating potential mechanistic links and identifying novel biomarkers or therapeutic targets.

## 2. Methods

### 2.1. Study Design

**Figure 1:**
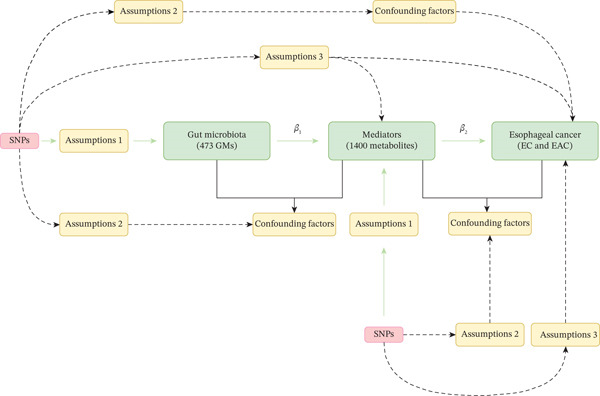
Study design and overview of our study. EC, esophageal cancer; EAC, esophageal adenocarcinoma; SNP, single‐nucleotide polymorphism.

The present MR study analyzed a total of 1873 categories of modifiable factors, comprising 473 GMs and 1400 metabolite traits. The study was conducted in three stages, as illustrated in Figure 1, which outlines the study design. In the first stage, we assessed the causal effects (*β*_all_) of each GM category on EC and EAC using two‐sample Mendelian randomization (TSMR). Subsequently, we examined the causal effects (*β*_2_) of each metabolite category on EC and EAC, also employing TSMR. Finally, we investigated potential mediators in the relationships between GMs and EC, EAC, and conducted a mediation analysis to quantify the impact of the mediators (mediation effect: *β*_1_?*β*_2_, where *β*_2_ represents the effect of GMs on metabolites) on the associations between GMs and EC and EAC.

### 2.2. Data Source

The GM genetic data were obtained from the latest genome‐wide association study (GWAS) summary, which included analyses of 2801 microbial taxa and 7,967,866 human genetic variants from 5959 individuals in the FR02 cohort [21]. This GWAS summary included a total of 473 GM. Additionally, GWAS data on 1400 metabolites were obtained from the study by Chen et al. [22], providing a more comprehensive dataset than the 486 metabolite GWAS data reported by Shin et al. [19]. Thus far, this represents the most extensive metabolite‐related GWAS dataset available. The GWAS summary statistics have been archived in the GWAS catalog (https://www.ebi.ac.uk/gwas/). The dataset originates from 8299 individuals participating in the Canadian Longitudinal Study on Aging (CLSA) cohort.

The summary data for the GWAS of EC were sourced from the 11th version of the Finngen consortium. This prospective cohort study involved screening patients using International Classification of Diseases diagnosis codes specific to EC. The dataset pertaining to EAC utilized in this research was obtained from the GWAS database (https://www.ebi.ac.uk/gwas/). The EAC dataset comprises information from 21,271 participants of European ancestry.

This research involves a secondary analysis of publicly accessible GWAS summary statistics. Ethical approvals were secured for each original GWAS study, and no data at the individual level was used. Therefore, obtaining new ethical review board approval was not required. All GWAS summary statistics used in this study were derived predominantly from participants of European ancestry, as reported in the original GWAS publications.

### 2.3. IV Selection

The MR analysis evaluates the influence of a predictor variable on an outcome variable. For an IV to be considered valid, it must fulfill three fundamental assumptions: (a) it must be correlated with the exposure, known as the “relevance” assumption; (b) it should be independent of the outcome, conditional on the exposure, referred to as the “exclusion restriction” assumption; and (c) it must be independent of all confounding variables, whether observed or unobserved, which is termed the “exchangeability” assumption [23, 24]. If an IV is correlated with a confounding factor that influences both the exposure and the outcome, the underlying assumptions may be compromised, potentially resulting in biases and erroneous conclusions. Thus, genetic IVs for GM, metabolites, EC, and EAC were developed based on the following criteria [25]: (a) *r* ^2^ measure of linkage disequilibrium (LD) among IVs < 0.001 at a 10000‐kb window, (b) *p* < 1 × 10^−5^, (c) *F* − statistics > 10, and (d) nonpalindromic single‐nucleotide polymorphisms (SNPs).

### 2.4. MR Analyses

The primary MR analyses were performed using the inverse‐variance weighted (IVW) method, which is widely regarded as the benchmark approach in MR studies. This technique applies a meta‐analytic framework to combine the ratio estimates of individual SNPs, weighting each by the inverse of its variance, thereby yielding a pooled estimate of the causal effect of the exposure on the outcome [26, 27]. The ratio estimate for each SNP reflects its effect on the outcome per unit increase in the exposure, assuming a log‐linear relationship between exposure and outcome [28]. The IVW estimator is most robust when all IVs are valid—that is, they satisfy the three core assumptions of MR: relevance, independence, and exclusion restriction [29].

To strengthen the robustness of our findings and account for potential violations of IV assumptions, we complemented the IVW method with several sensitivity analyses, including MR‐Egger regression, weighted median, weighted mode, and simple mode estimators. These alternative approaches offer varying levels of tolerance to invalid instruments and horizontal pleiotropy, enhancing the credibility of causal inference in the presence of potential bias.

### 2.5. Mediation Analysis

To assess the potential mediating role of metabolites in the causal pathway from GM to EC and EAC, we conducted a two‐step MR mediation analysis. In this framework, a mediating effect is supported when both the total and indirect effects point in the same direction, indicating consistent causal flow through the intermediate trait.

The proportion of the effect mediated by metabolites was calculated using the following formula:

Mediation proportion=11−direct effecttotal effect=−βall−β1×β2βall

where *β*_all_ denotes the total effect of GM on EC/EAC, *β*_1_ represents the effect of GM on the metabolite, and *β*_2_ denotes the effect of the metabolite on EC/EAC. This approach quantifies the extent to which metabolites account for the overall GM–cancer association.

### 2.6. Genetic Risk Scores (GRSs)

To further validate the causal relationships identified through MR, we conducted a supplementary analysis using GRSs. This analysis was implemented in R (version for Windows), utilizing the gtx package (v0.0.8), specifically the grs.summary function. This function performs GRS estimation based on summary‐level data from GWAS, using an approach analogous to regressing the outcome on a weighted additive GRS [30, 31]. For sets of uncorrelated SNPs, the causal effect estimate (*α*) was derived using the approximation:

α≈∑ωβseβ−2∑ω2seβ−2

and its standard error (se_*α*) was estimated as follows:

seα≈1∑ω2seβ−2

Here, *ω* denotes the SNP‐specific effect on the exposure (e.g., GM or metabolite), *β* represents the SNP effect on the outcome (EC or EAC), and se_*β* is the corresponding standard error [30, 32]. This method enables robust effect estimation when aggregating information across multiple genetic variants, thereby enhancing the interpretability and reliability of causal inference.

### 2.7. Sensitivity Analysis

To evaluate the robustness of our MR findings, we conducted multiple sensitivity analyses addressing potential horizontal pleiotropy and heterogeneity. Horizontal pleiotropy was assessed using the MR‐Egger intercept test and the Global test. A *p* value > 0.05 in both tests indicated no significant directional pleiotropy, supporting the validity of the IVs.

Cochran′s *Q* test was employed to evaluate heterogeneity among SNP‐specific causal estimates. If the *p* value from this test was < 0.05, the IVW method was applied under a multiplicative random‐effects model to account for between‐instrument variability.

In addition, we performed a leave‐one‐out analysis to determine whether any single IV disproportionately influenced the causal estimate. The strength of instrument–exposure associations was quantified using the *F* statistic, with higher values indicating stronger instrument relevance [33].

Effect estimates are reported as odds ratios (ORs) or beta coefficients, along with 95% confidence intervals (CIs). Statistical significance was defined as a *p* value < 0.05. All analyses were conducted in R Version 4.1.2, using the packages “MendelianRandomization,” “TwoSampleMR,” and “gtx” [34].

## 3. Results

### 3.1. Genetic Instruments Selection for GM: Metabolites

We systematically selected genetic instruments from GWAS datasets encompassing 473 GM traits and 1400 circulating metabolites, applying stringent inclusion criteria. All IVs demonstrated *F* statistics greater than 10, thereby minimizing the risk of weak instrument bias. The full list of selected IVs for GM and metabolites is provided in Tables S1 and S2, respectively.

### 3.2. Causal Effects of GM on EC: EAC

**Figure 2:**
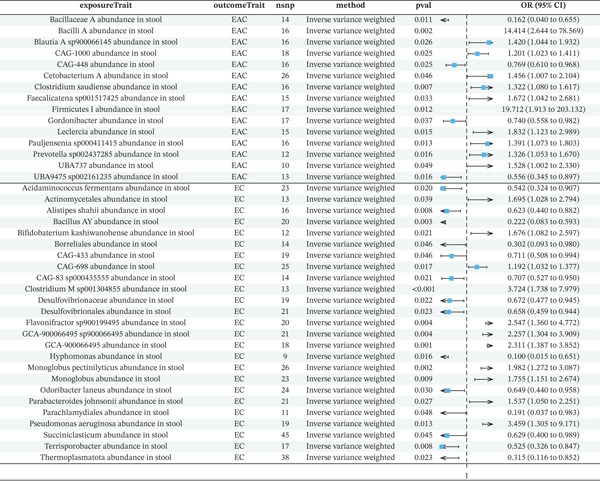
Forest plot of MR study based on the IVW method from GMs to EC and EAC. IVW: inverse‐variance weighted; EC, esophageal cancer; EAC, esophageal adenocarcinoma; GM, gut microbiota; snp, single‐nucleotide polymorphism.

A total of 25 GMs were found to be associated with EC (Figures 2 and S1). Detailed SNP information for these 25 GMs is presented in Table S3. As illustrated in Figure 2 , MR analysis indicated that genetic prediction of 11 GMs (*Clostridium M sp001304855*, *Pseudomonas aeruginosa*, *Flavonifractor sp900199495*, *GCA−900066495*, *GCA−900066495 sp900066495*, *Monoglobus pectinilyticus*, *Monoglobus*, Actinomycetales, *Bifidobacterium kashiwanohense*, *Parabacteroides johnsonii* and *P. aeruginosa*) was associated with an increased risk of EC (OR > 1, *p* < 0.05). Conversely, 14GMs (*CAG-433*, *CAG-83* *sp000435555*, *Desulfovibrionaceae*, *Desulfovibrionales*, *Succiniclasticum*, *Alistipes shahi*, *Acidaminococcus fermentans*, *Terrisporobacter*, *Thermoplasmatota*, *Borreliale*, *Bacillus AY*, *Parachlamydiales*, *Odoribacter laneus and Hyphomonas*) significantly decreased the risk of EC (OR < 1, *p* < 0.05).

Additionally, a total of 15 GMs showed an association with EAC (Figures 2 and S1). Detailed SNPs information for 15 GMs is shown in Table S3. As shown in Figure 2, MR analysis suggested that genetic prediction of 11 GMs (Firmicutes I, Bacilli A, *Leclerci*, *Faecalicatena sp001517425*, *UBA737*, *Cetobacterium*, *Blautia A sp900066145*, *Pauljensenia sp00041141*, *Prevotella sp00243728*, *Clostridium saudiense*, and *CAG-1000*) was associated with an increased risk of EAC (OR > 1, *p* < 0.05). Meanwhile, four GMs (*CAG-448*, *Gordonibacter*, *UBA9475 sp002161235*, and Bacillaceae *A*) significantly decreased the risk of EAC (OR < 1, *p* < 0.05).

As for sensitivity analysis, Cochrane′s *Q* tests, Global test, and MR‐Egger regression intercept analysis demonstrated no heterogeneity or horizontal pleiotropy in the association of GMs with EC and EAC MR (Table 1). Furthermore, the leave‐one‐out plot indicated that no single SNP predominantly influenced the genetic associations regarding GMs with EC and EAC MR (Figure S2).

**Table 1:** Heterogeneity and horizontal pleiotropy test of GMs and EC/EAC MR analyses.

Exposure	Outcome	MR‐Egger intercept *p*	Global test intercept *p*	Cochran′s *Q*	Cochran′s *Q* *p*
Bacillaceae A abundance in stool	EAC	0.083	0.254	16.707	0.213
Bacilli A abundance in stool	EAC	0.546	0.990	5.311	0.989
*Blautia* A sp900066145 abundance in stool	EAC	0.326	0.397	15.911	0.388
CAG‐1000 abundance in stool	EAC	0.700	0.740	13.650	0.692
CAG‐448 abundance in stool	EAC	0.184	0.347	16.827	0.329
*Cetobacterium* A abundance in stool	EAC	0.366	0.876	17.051	0.880
*Clostridium saudiense* abundance in stool	EAC	0.633	0.835	10.419	0.793
*Faecalicatena* sp001517425 abundance in stool	EAC	0.346	0.650	11.599	0.639
Firmicutes I abundance in stool	EAC	0.313	0.942	8.740	0.924
*Gordonibacter* abundance in stool	EAC	0.738	0.614	14.393	0.569
*Leclercia* abundance in stool	EAC	0.924	0.676	11.726	0.628
*Pauljensenia* sp000411415 abundance in stool	EAC	0.213	0.520	14.917	0.457
*Prevotella* sp002437285 abundance in stool	EAC	0.904	0.730	8.217	0.694
UBA737 abundance in stool	EAC	0.680	0.232	12.975	0.164
UBA9475 sp002161235 abundance in stool	EAC	0.669	0.801	8.210	0.769
*Acidaminococcus fermentans* abundance in stool	EC	0.975	0.420	22.621	0.423
Actinomycetales abundance in stool	EC	0.988	0.510	11.522	0.485
*Alistipes shahii* abundance in stool	EC	0.176	0.350	17.114	0.312
*Bacillus* AY abundance in stool	EC	0.801	0.465	19.176	0.446
*Bifidobacterium kashiwanohense* abundance in stool	EC	0.860	0.269	14.338	0.215
Borreliales abundance in stool	EC	0.452	0.991	3.813	0.993
CAG‐433 abundance in stool	EC	0.761	0.975	7.774	0.982
CAG‐698 abundance in stool	EC	0.556	0.597	22.097	0.573
CAG‐83 sp000435555 abundance in stool	EC	0.321	0.227	16.803	0.208
*Clostridium* M sp001304855 abundance in stool	EC	0.863	0.817	7.957	0.788
Desulfovibrionaceae abundance in stool	EC	0.279	0.650	15.379	0.636
Desulfovibrionales abundance in stool	EC	0.214	0.613	17.468	0.622
*Flavonifractor* sp900199495 abundance in stool	EC	0.464	0.369	20.760	0.350
GCA‐900066495 sp900066495 abundance in stool	EC	0.322	0.323	22.518	0.313
GCA‐900066495 abundance in stool	EC	0.143	0.422	17.896	0.395
*Hyphomonas* abundance in stool	EC	0.783	0.478	7.969	0.437
*Monoglobus pectinilyticus* abundance in stool	EC	0.675	0.156	32.658	0.140
*Monoglobus* abundance in stool	EC	0.590	0.206	28.082	0.173
*Odoribacter laneus* abundance in stool	EC	0.745	0.837	16.616	0.828
*Parabacteroides johnsonii* abundance in stool	EC	0.241	0.331	21.859	0.348
Parachlamydiales abundance in stool	EC	0.512	0.427	10.332	0.412
*Pseudomonas aeruginosa* abundance in stool	EC	0.393	0.232	22.270	0.220
*Succiniclasticum* abundance in stool	EC	0.502	0.465	44.532	0.449
*Terrisporobacter* abundance in stool	EC	0.646	0.868	10.007	0.866
Thermoplasmatota abundance in stool	EC	0.762	0.324	41.220	0.291

Abbreviations: EAC, esophageal adenocarcinoma; EC, esophageal cancer.

### 3.3. Bidirectional Causal Effects of EC and EAC on GMs

To eliminate the possibility of reverse causality, we performed a reverse MR analysis examining the relationships between EC and EAC with GM. Following the established screening criteria, we ultimately identified 20 SNPs as IVs for EC and 22 SNPs for EAC. The MR results indicated a reverse causal relationship between one GM and EC (*Pseudomonas aeruginosa abundance in stool*: OR: 1.0105, 95% CI: 1.0004–1.0208, *p* = 0.0421), whereas no reverse causal relationship was identified between GMs and EAC (Table S4).

### 3.4. Causal Effects of Metabolites on EC, EAC

**Figure 3:**
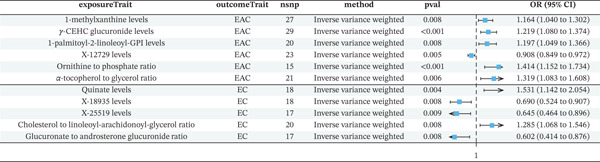
Forest plot of MR study based on the IVW method from metabolites to EC and EAC. IVW: inverse‐variance weighted; EC, esophageal cancer; EAC, esophageal adenocarcinoma; snp, single‐nucleotide polymorphism.

A total of five metabolites were associated with EC (Figure 3). Detailed information regarding the SNPs related to these five metabolites is presented in Table S5. As shown in Figure 3, MR analysis suggested that genetic prediction of 2 metabolites (*Quinate levels, cholesterol to linoleoyl-arachidonoyl-glycerol ratio*) was associated with an increased risk of EC (OR > 1, *p* < 0.05). Conversely, three metabolites (*X-18935 levels, X-25519 levels, and glucuronate to androsterone glucuronide ratio*) significantly decreased the risk of EC (OR < 1, *p* < 0.05).

In addition, a total of six metabolites were associated with EAC (Figure 3). The detailed SNPs information for six metabolites is provided in Table S5. As illustrated in Figure 3, MR analysis revealed that the genetic prediction of 5 metabolites (*1-methylxanthine levels, Gamma-CEHC glucuronide levels, 1-palmitoyl-2-linoleoyl-GPI levels, ornithine-to-phosphate ratio, alpha-tocopherol-to-glycerol ratio*) was related to an increased risk of EAC (OR > 1, *p* < 0.05). Notably, one metabolite (*X-12729 levels*) significantly decreased the risk of EAC (OR < 1, *p* < 0.05).

As for sensitivity analysis, Cochrane′s *Q* tests, Global test, and MR‐Egger regression intercept analysis demonstrated no heterogeneity or horizontal pleiotropy in the association of metabolites with EC and EAC MR (Table 2). Furthermore, the leave‐one‐out plot indicated that no single SNP predominantly influenced the genetic associations regarding metabolites with EC and EAC MR (Figure S3).

**Table 2:** Heterogeneity and horizontal pleiotropy test of metabolites and EC/EAC MR analyses.

Exposure	Outcome	MR‐Egger intercept *p*	Global test intercept *p*	Cochran′s *Q*	Cochran′s *Q* *p*
1‐methylxanthine levels	EAC	0.984	0.745	21.945	0.692
Gamma‐CEHC glucuronide levels	EAC	0.881	0.445	27.784	0.476
1‐palmitoyl‐2‐linoleoyl‐GPI levels	EAC	0.743	0.540	18.360	0.499
X‐12729 levels	EAC	0.458	0.544	23.280	0.386
Ornithine‐to‐phosphate ratio	EAC	0.530	0.743	10.557	0.720
Alpha‐tocopherol‐to‐glycerol ratio	EAC	0.990	0.692	16.408	0.691
Quinate levels	EC	0.154	0.245	23.519	0.215
X‐18935 levels	EC	0.463	0.342	22.158	0.332
X‐25519 levels	EC	0.900	0.697	15.231	0.646
Cholesterol to linoleoyl‐arachidonoyl‐glycerol ratio	EC	0.955	0.926	13.392	0.894
Glucuronate‐to‐androsterone glucuronide ratio	EC	0.930	0.131	22.859	0.118

Abbreviations: EAC, esophageal adenocarcinoma; EC, esophageal cancer.

### 3.5. Mediation Analysis

**Figure 4:**
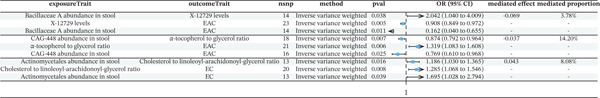
Forest plot of mediated MR study based on the IVW method. IVW: inverse‐variance weighted; EC, esophageal cancer; EAC, esophageal adenocarcinoma; snp, single‐nucleotide polymorphism.

In this study, GM and metabolites all had causal effects on EC and EAC. It seemed that metabolites played a mediating effect in the pathway from GM to EC and EAC. The conditions for a mediating effect require a significant association between GM and metabolites, as well as a consistent directional alignment of both the total effects and mediated effects. Our results indicated that, among the GM and metabolites associated with EC and EAC, one metabolite demonstrated a mediating effect in the relationship between GM and EC (*Actinomycetales abundance in stool–cholesterol to linoleoyl-arachidonoyl-glycerol ratio–EC*, Figure 4), and the mediated effect of *cholesterol to linoleoyl-arachidonoyl-glycerol ratio* is 0.043 and the mediated proportion is 8.08%. Furthermore, two metabolites exhibited mediating effects in the association between GM and EAC (*Bacillaceae–X-12729 levels–EAC*; *CAG-448–alpha*‐*tocopherol-to-glycerol ratio–EAC*, Figure 4), and the mediated effect of *X-12729*, *alpha-tocopherol-to-glycerol ratio* is −0.069 and −0.037 and the mediated proportion is 3.78% and 14.20%, respectively. Therefore, our research demonstrated that metabolites serve as intermediaries in the pathway linking GM to EC and EAC.

### 3.6. GRS Validation

To enhance the reliability of our results, we employed the GRS method to validate the IVW findings. As presented in Table 3 and Table 4, there was a strong concordance between the GRS and IVW methods, leading to the identification of 25 GMs‐related EC, 6 metabolites associated with EC, 15 GMs‐related EAC, and 3 metabolites linked to EAC. Furthermore, the metabolites exhibited mediating effects within the GM and EC/EAC pathways and were further validated by GRS methods (Table 5).

**Table 3:** Results of GMs and EC/EAC GRS validation analysis.

Exposure	Outcome	Methods	Beta	Se	*p*	*Q*	*p*_*Q*_
Bacillaceae A abundance in stool	EAC	GRS	−1.821	0.629	0.004	16.707	0.213
Bacilli A abundance in stool	EAC	GRS	2.668	0.865	0.002	5.311	0.989
Blautia A sp900066145 abundance in stool	EAC	GRS	0.351	0.152	0.021	15.911	0.388
CAG‐1000 abundance in stool	EAC	GRS	0.183	0.082	0.025	13.650	0.692
CAG‐448 abundance in stool	EAC	GRS	−0.263	0.111	0.018	16.827	0.329
*Cetobacterium* A abundance in stool	EAC	GRS	0.376	0.188	0.046	17.051	0.880
*Clostridium saudiense* abundance in stool	EAC	GRS	0.279	0.103	0.007	10.419	0.793
*Faecalicatena* sp001517425 abundance in stool	EAC	GRS	0.514	0.241	0.033	11.599	0.639
Firmicutes I abundance in stool	EAC	GRS	2.981	1.190	0.012	8.740	0.924
*Gordonibacter* abundance in stool	EAC	GRS	−0.301	0.144	0.037	14.393	0.569
*Leclercia* abundance in stool	EAC	GRS	0.606	0.250	0.015	11.726	0.628
*Pauljensenia* sp000411415 abundance in stool	EAC	GRS	0.330	0.132	0.013	14.917	0.457
*Prevotella* sp002437285 abundance in stool	EAC	GRS	0.283	0.118	0.016	8.217	0.694
UBA737 abundance in stool	EAC	GRS	0.424	0.179	0.018	12.975	0.164
UBA9475 sp002161235 abundance in stool	EAC	GRS	−0.586	0.244	0.016	8.210	0.769
*Acidaminococcus fermentans* abundance in stool	EC	GRS	−0.612	0.259	0.018	22.621	0.423
Actinomycetales abundance in stool	EC	GRS	0.528	0.255	0.039	11.522	0.485
*Alistipes shahii* abundance in stool	EC	GRS	−0.473	0.166	0.004	17.114	0.312
*Bacillus* AY abundance in stool	EC	GRS	−1.503	0.498	0.003	19.176	0.446
*Bifidobacterium kashiwanohense* abundance in stool	EC	GRS	0.516	0.196	0.008	14.338	0.215
Borreliales abundance in stool	EC	GRS	−1.196	0.600	0.046	3.813	0.993
CAG‐433 abundance in stool	EC	GRS	−0.341	0.171	0.046	7.774	0.982
CAG‐698 abundance in stool	EC	GRS	0.176	0.074	0.017	22.097	0.573
CAG‐83 sp000435555 abundance in stool	EC	GRS	−0.346	0.132	0.009	16.803	0.208
*Clostridium* M sp001304855 abundance in stool	EC	GRS	1.315	0.389	0.001	7.957	0.788
Desulfovibrionaceae abundance in stool	EC	GRS	−0.398	0.174	0.022	15.379	0.636
Desulfovibrionales abundance in stool	EC	GRS	−0.418	0.184	0.023	17.468	0.622
*Flavonifractor* sp900199495 abundance in stool	EC	GRS	0.935	0.306	0.002	20.760	0.350
GCA‐900066495 sp900066495 abundance in stool	EC	GRS	0.814	0.264	0.002	22.518	0.313
GCA‐900066495 abundance in stool	EC	GRS	0.838	0.254	0.001	17.896	0.395
*Hyphomonas* abundance in stool	EC	GRS	−2.303	0.956	0.016	7.969	0.437
*Monoglobus pectinilyticus* abundance in stool	EC	GRS	0.684	0.198	0.001	32.658	0.140
*Monoglobus* abundance in stool	EC	GRS	0.562	0.190	0.003	28.082	0.173
*Odoribacter laneu*s abundance in stool	EC	GRS	−0.432	0.199	0.030	16.616	0.828
*Parabacteroides johnsonii* abundance in stool	EC	GRS	0.430	0.186	0.021	21.859	0.348
Parachlamydiales abundance in stool	EC	GRS	−1.655	0.822	0.044	10.332	0.412
*Pseudomonas aeruginosa* abundance in stool	EC	GRS	1.241	0.447	0.006	22.270	0.220
*Succiniclasticum* abundance in stool	EC	GRS	−0.464	0.230	0.044	44.532	0.449
*Terrisporobacter* abundance in stool	EC	GRS	−0.644	0.244	0.008	10.007	0.866
Thermoplasmatota abundance in stool	EC	GRS	−1.156	0.481	0.016	41.220	0.291

Abbreviations: EAC, esophageal adenocarcinoma; EC, esophageal cancer; GRS, genetic risk scores.

**Table 4:** Results of metabolites and EC/EAC GRS validation analysis.

Exposure	Outcome	Methods	Beta	Se	*p*	*Q*	*p*_*Q*_
1‐Methylxanthine levels	EAC	GRS	0.152	0.057	0.008	21.945	0.692
Gamma‐CEHC glucuronide levels	EAC	GRS	0.198	0.061	0.001	27.784	0.476
1‐Palmitoyl‐2‐linoleoyl‐GPI levels	EAC	GRS	0.180	0.068	0.008	18.360	0.499
X‐12729 levels	EAC	GRS	−0.096	0.034	0.004	23.280	0.386
Ornithine‐to‐phosphate ratio	EAC	GRS	0.346	0.104	0.001	10.557	0.720
Alpha‐tocopherol‐to‐glycerol ratio	EAC	GRS	0.277	0.101	0.006	16.408	0.691
Quinate levels	EC	GRS	0.426	0.136	0.002	20.621	0.244
X‐18935 levels	EC	GRS	−0.372	0.123	0.003	21.794	0.193
X‐25519 levels	EC	GRS	−0.439	0.168	0.009	13.109	0.665
Cholesterol to linoleoyl‐arachidonoyl‐glycerol ratio	EC	GRS	0.250	0.094	0.008	10.465	0.941
Glucuronate to androsterone glucuronide ratio	EC	GRS	−0.508	0.160	0.002	22.859	0.118

Abbreviations: EAC, esophageal adenocarcinoma; EC, esophageal cancer; GRS, genetic risk scores.

**Table 5:** Results of GMs–metabolites–EC/EAC GRS mediation validation analysis.

Exposure	Outcome	Methods	Beta	Se	*p*	*Q*	*p*_*Q*_
Bacillaceae A abundance in stool	X‐12729 levels	GRS	0.714	0.344	0.038	10.467	0.655
X‐12729 levels	EAC	GRS	−0.096	0.034	0.004	23.280	0.386
Bacillaceae A abundance in stool	EAC	GRS	−1.821	0.629	0.004	16.707	0.213
CAG‐448 abundance in stool	Alpha‐tocopherol‐to‐glycerol ratio	GRS	−0.135	0.050	0.007	14.264	0.648
Alpha‐tocopherol‐to‐glycerol ratio	EAC	GRS	0.277	0.101	0.006	16.408	0.691
CAG‐448 abundance in stool	EAC	GRS	−0.263	0.111	0.018	16.827	0.329
Actinomycetales abundance in stool	Cholesterol to linoleoyl‐arachidonoyl‐glycerol ratio	GRS	0.170	0.072	0.018	3.184	0.994
Cholesterol to linoleoyl‐arachidonoyl‐glycerol ratio	EC	GRS	0.250	0.094	0.008	10.465	0.941
Actinomycetales abundance in stool	EC	GRS	0.528	0.255	0.039	11.522	0.485

Abbreviations: EAC, esophageal adenocarcinoma; EC, esophageal cancer; GMs, gut microbiotas; GRS, genetic risk scores.

## 4. Discussions

The pathogenesis of EC remains incompletely understood. Observational studies have suggested complex interactions among genetic predisposition, environmental exposures, and lifestyle behaviors in its development. An increasing body of evidence highlights causal links between genetic variants and multiple malignancies, including colorectal, gastric, lung, prostate, and ECs [35–39]. Concurrently, GM has emerged as a pivotal factor in cancer biology, showing promising implications for tumor immunotherapy [40, 41]. Several studies have also indicated that microbial dysbiosis may contribute to the development of EC [42]. Moreover, host–microbiome cometabolites—encompassing both substrates and metabolic end‐products—have been linked to EC pathogenesis [15, 16]. Despite these findings, the causal relationship between GM and EC and the mechanistic role of metabolites in this axis has been difficult to establish due to confounding in observational studies and the limitations of traditional epidemiological designs.

To address these gaps, we employed a two‐step MR framework, integrating mediation analysis to systematically investigate whether circulating metabolites mediate the causal effects of GM on EC and its major subtype, EAC. Using large‐scale GWAS datasets encompassing 473 GM traits and 1400 metabolites, we rigorously selected valid IVs and performed sensitivity analyses to minimize heterogeneity and horizontal pleiotropy. Our approach provides robust causal inference beyond conventional observational strategies.

We also identified five metabolites causally linked to EC—two risk‐associated (e.g., quinate levels and cholesterol to linoleoyl‐arachidonoyl‐glycerol ratio) and three protective (e.g., X‐18935, X‐25519, and glucuronate to androsterone glucuronide ratio). In EAC, six metabolites were implicated, of which five were associated with increased risk (e.g., 1‐methylxanthine, gamma‐CEHC glucuronide, and ornithine‐to‐phosphate ratio) and one (X‐12729) was protective. Notably, mediation analysis revealed several key metabolite–GM–cancer pathways: cholesterol to linoleoyl‐arachidonoyl‐glycerol ratio mediated the effect of Actinomycetales on EC (mediation proportion~8.08%), X‐12729 mediated the effect of Bacillaceae on EAC (~3.78%), and alpha‐tocopherol‐to‐glycerol ratio mediated the association between CAG*-*448 and EAC (~14.20%). These findings not only support causal associations between GM and EC/EAC but also identify metabolites as mechanistic intermediaries, providing new insights into host–microbiome–metabolite interactions in carcinogenesis.

Our study offers several strengths. First, by leveraging the MR framework, we minimized confounding and reverse causation, enhancing the credibility of causal inference. Second, the GWAS datasets were primarily derived from European populations, reducing potential bias due to population stratification. Third, sensitivity analyses—including MR‐Egger intercept, Global test, and Cochran′s *Q* test—showed no evidence of significant pleiotropy or heterogeneity, whereas leave‐one‐out analysis indicated no undue influence from individual SNPs. Lastly, results from the GRS analysis were consistent with those from the IVW approach, underscoring the robustness of our findings. Nonetheless, several limitations merit consideration. Our analyses were restricted to individuals of European ancestry, and further validation in other ethnic populations is needed. In addition, although our study provides genetic evidence supporting GM–metabolite–EC pathways, mechanistic studies at the cellular and molecular levels are required to elucidate the biological underpinnings of these associations.

## 5. Conclusion

In summary, this study provides comprehensive genetic evidence supporting the causal roles of GM and circulating metabolites in the development of EC and its subtype, EAC. Importantly, we demonstrate that specific metabolites mediate the effects of GM on cancer risk, revealing a complex triadic interplay among host genetics, microbial composition, and metabolic environment. These findings contribute to a deeper understanding of EC pathogenesis and may inform future strategies for early detection, prevention, and personalized intervention.

**Nomenclature**
CRT: chemoradiotherapy
EC: esophageal cancer
EAC: esophageal adenocarcinoma
IVs: instrumental variables
GWAS: genome‐wide association studies
MR: Mendelian randomization
IVW: inverse‐variance weighted
GRS: genetic risk score
GM: gut microbiota
SNP: single‐nucleotide polymorphism.

## Author Contributions

All authors contributed significantly to the work. H.Y., Y.L., and T.Z.: conceptualization. Y.L. and T.Z.: data curation and resources. H.Y.: formal analysis, methodology, and software. T.Z., Z.X., and H.W.: investigation. Y.C.: supervision. S.Z.: validation. Y.L. and S.Z.: visualization. H.Y. and Y.C.: writing—original draft. All authors: writing—review and editing. H.Y., Y.L., and T.Z. contributed equally to this work and share first authorship.

## Funding

This study was supported by the National Natural Science Foundation of China (10.13039/501100001809) (82172076, 82472140); Open project of State Key Laboratory of Radiation Medicine and Radiation Protection (GZK12024018), Key Scientific Program of Jiangsu Provincial Health Commission (ZD2021033), Project of Medical New Technology Assistance of the Second Affiliated Hospital of Soochow University (23ZL004, 23ZL012), The Project of Capacity Enhancement of Institutional Clinical Trials in Suzhou (SLT2023030), The Project of Clinical Innovation and Interdisciplinary Translation of Soochow University (ML12301623), The Construction Project of High‐End Clinical Medical Technology Platform and Translational Base of Soochow University (ML12301123), and Discipline construction project of the Second Affiliated Hospital of Soochow University, Phase II (XKTJ‐XK202405).

## Disclosure

All authors approved the final version of the manuscript and agree to be accountable for all aspects of the work.

## Ethics Statement

All data utilized in this study are publicly accessible and have been obtained from research studies that have secured appropriate participant consent and ethical approval.

## Consent

All contributing authors have provided their consent for the publication of this work.

## Conflicts of Interest

The authors declare no conflicts of interest.

## Supporting Information

Additional supporting information can be found online in the Supporting Information section.

## Supporting information


**Supporting Information 1.** Figure S1: The scatterplots represent genetic IVs association between GM and EC/EAC. EC, esophageal cancer; EAC, esophageal adenocarcinoma.

**Supporting Information 2.** Figure S2: MR leave‐one‐out sensitivity analysis plots for GM on EA/EAC. EC, esophageal cancer; EAC, esophageal adenocarcinoma.

**Supporting Information 3.** Figure S3: MR leave‐one‐out sensitivity analysis plots for metabolites on EA/EAC. EC, esophageal cancer; EAC, esophageal adenocarcinoma.

**Supporting Information 4.** Table S1: Details of all the GM IVs that have been filtered. Table S2: Details of all the metabolites IVs that have been filtered. Table S3: Detailed SNP information on GMs associated with EC and EAC. Table S4: Reverse MR analysis results of EC and EAC to GM. Table S5: Detailed SNP information on metabolites associated with EC and EAC.

## Data Availability

This study is based entirely on publicly available GWAS summary statistics. Gut microbiota GWAS data were obtained from Qin et al. [21], metabolite GWAS data from Chen et al. [22], and EC/EAC GWAS data from FinnGen (v11) and the GWAS Catalog, respectively. Accession numbers/URLs are provided in the Methods.
