# Supplementary figures and images for: Gut Microbiome–Metabolite Interactions Contribute to Esophageal Cancer Risk: Evidence From Mendelian Randomization and Multiomics Integration
Source: Int J Genomics. 2026 Jun 8;2026:5967802. doi: 10.1155/ijog/5967802 (PMC13243870; doi:10.1155/ijog/5967802)

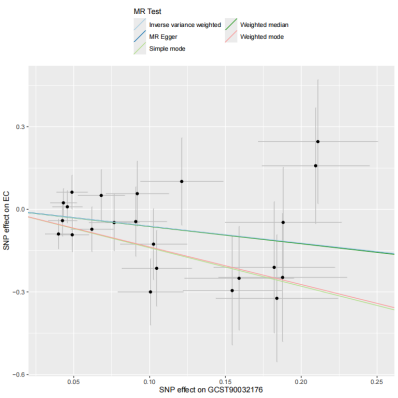

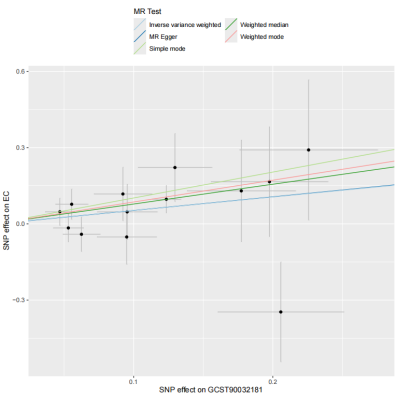

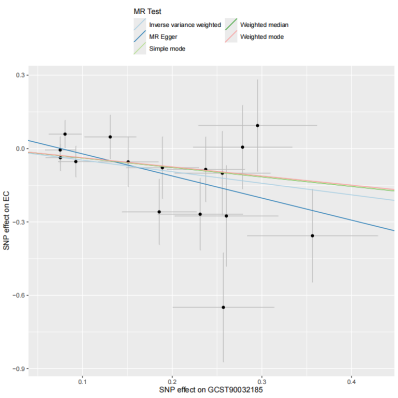

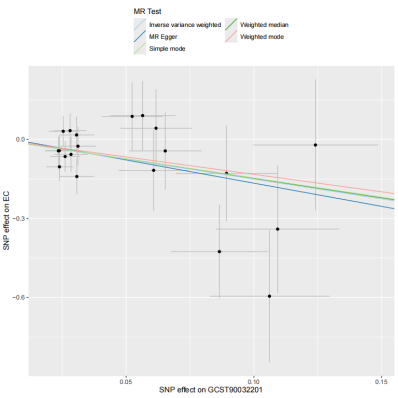

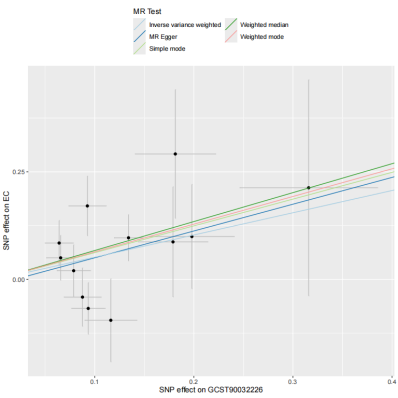

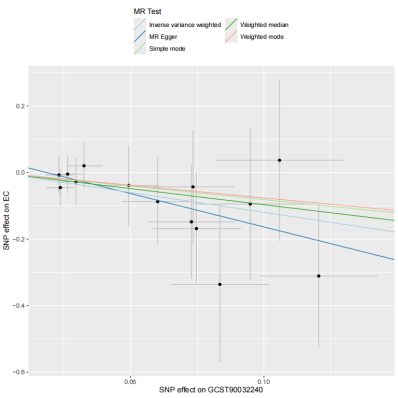

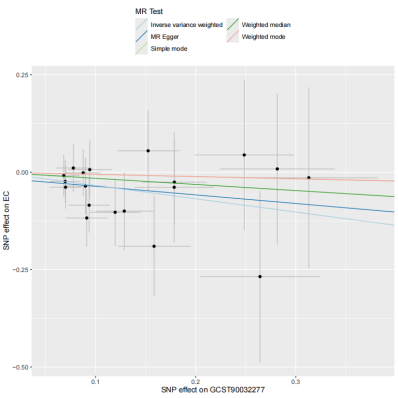

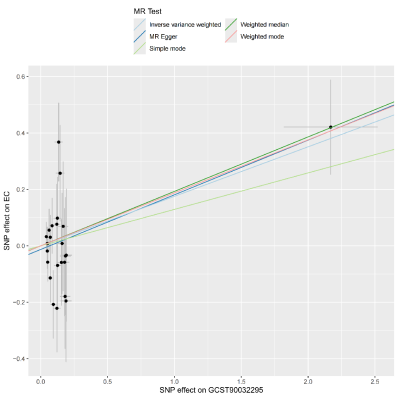

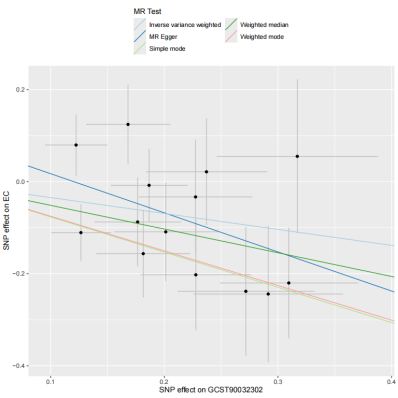

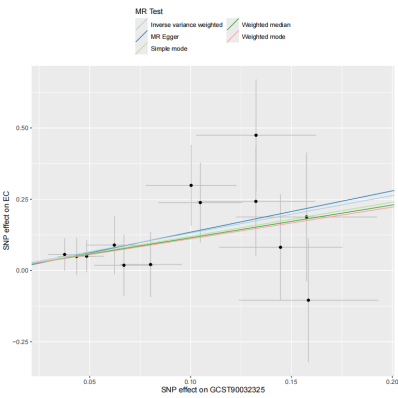

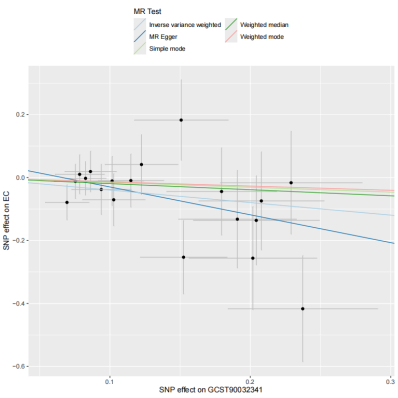

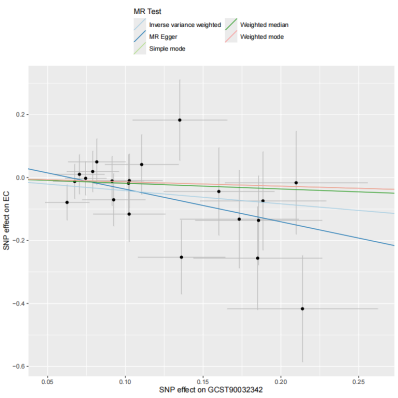

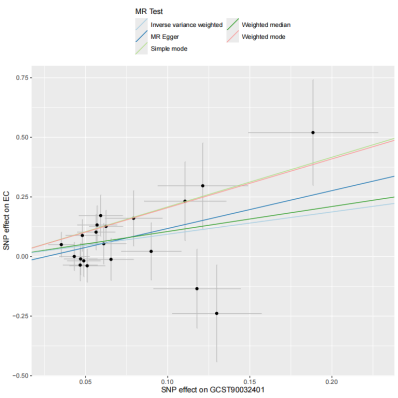

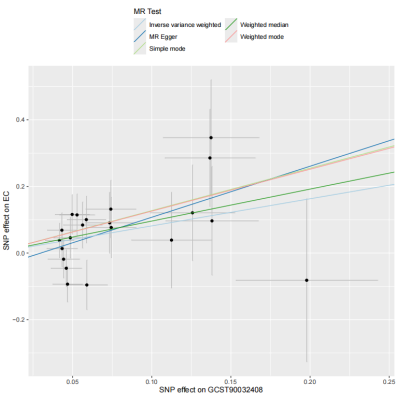

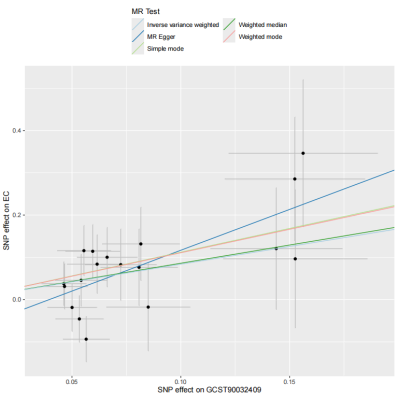

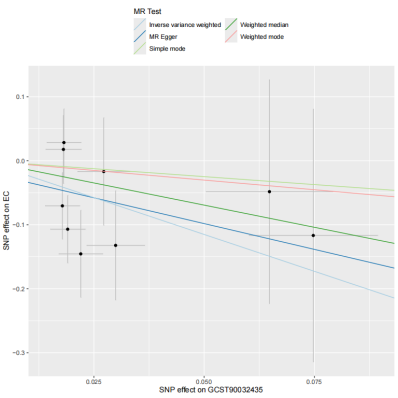

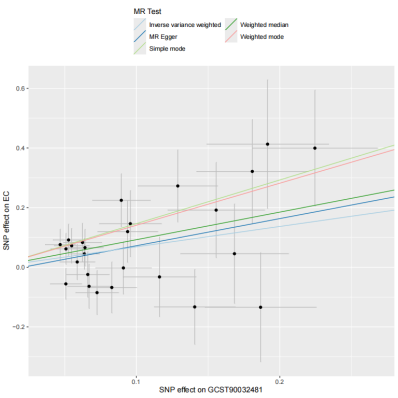

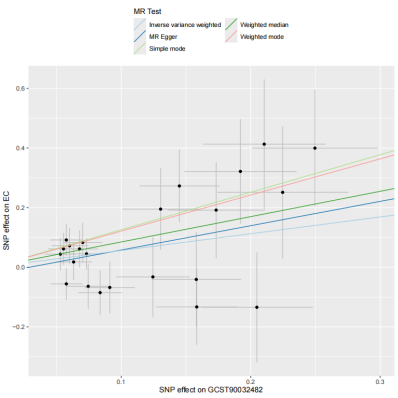

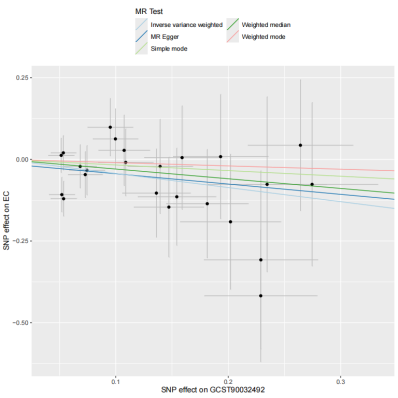

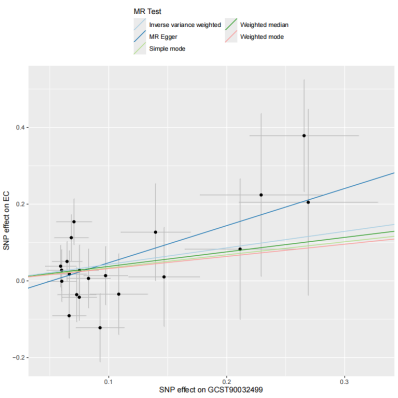

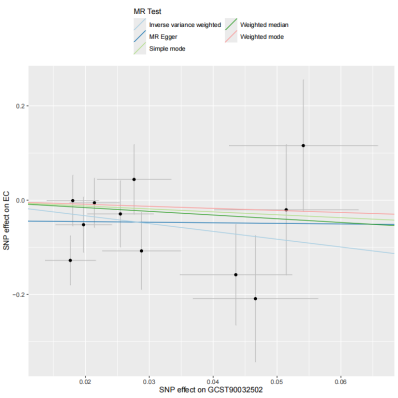

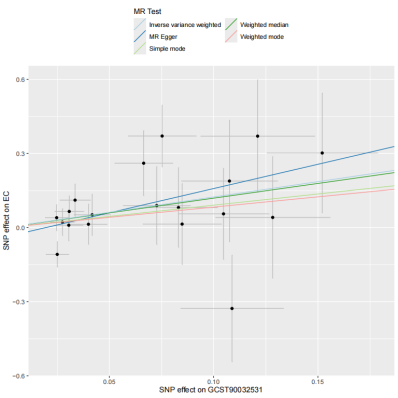

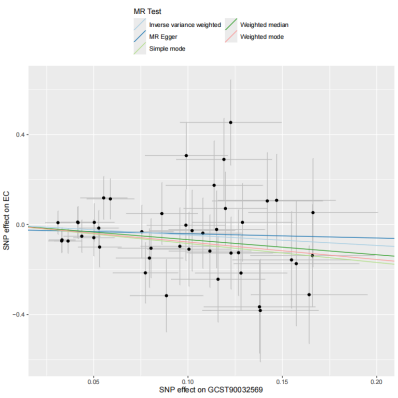

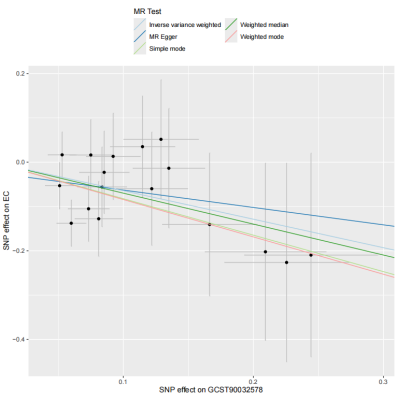

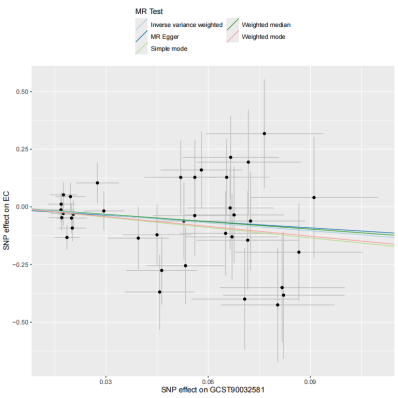

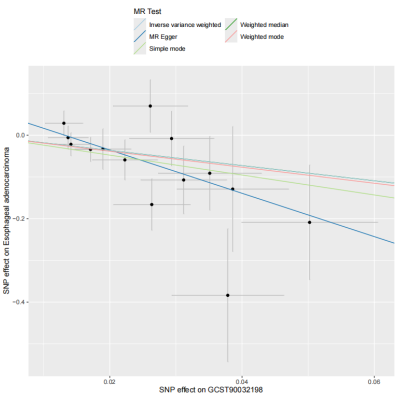

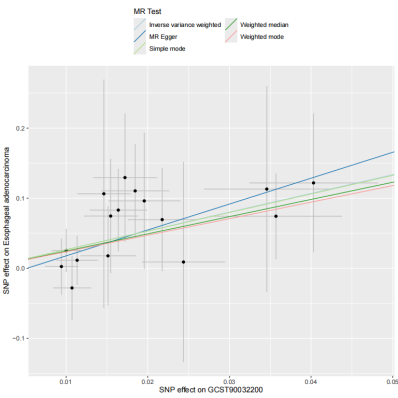

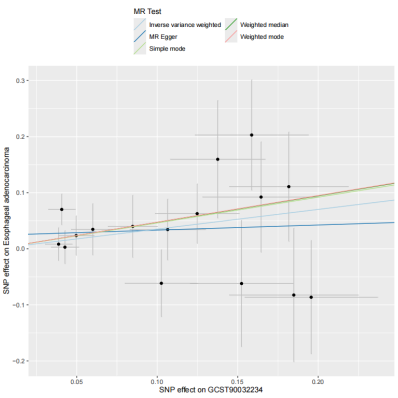

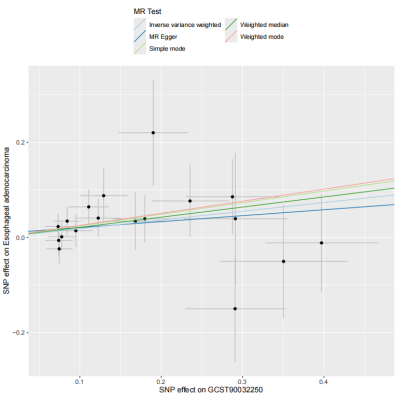

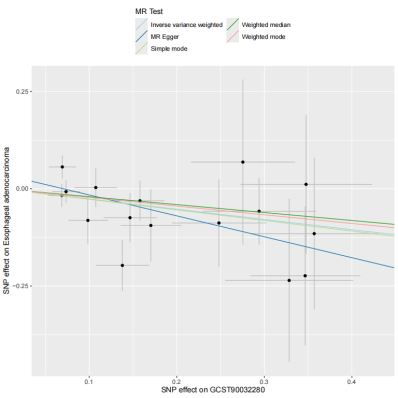

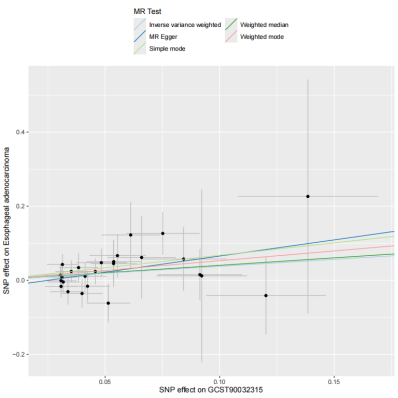

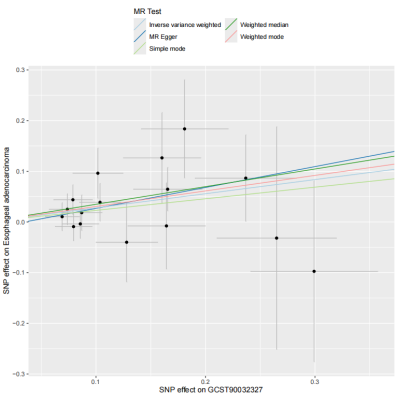

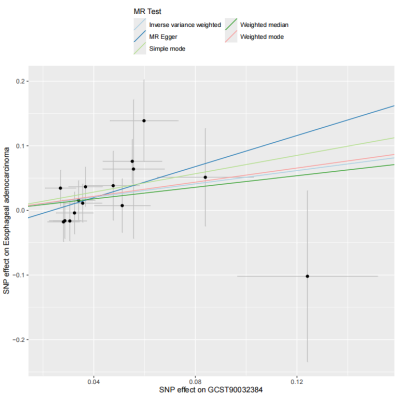

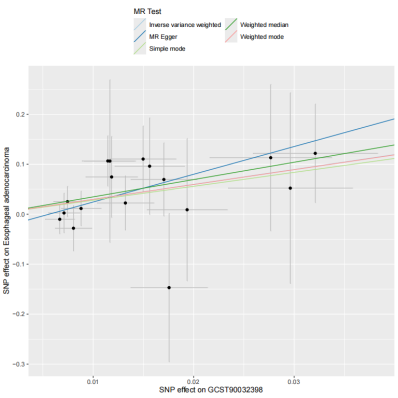

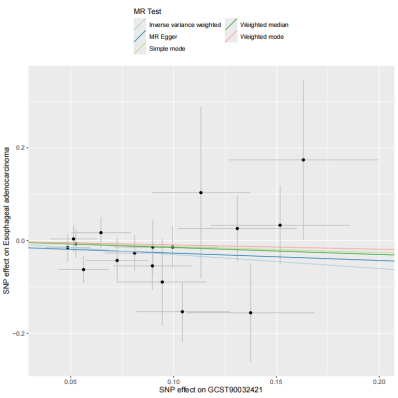

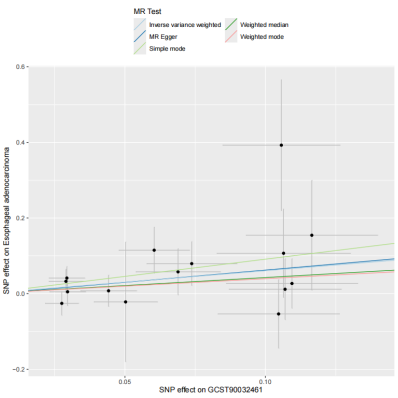

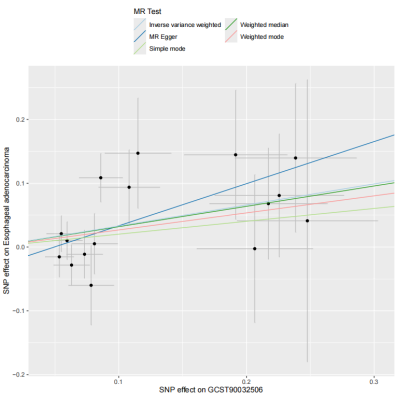

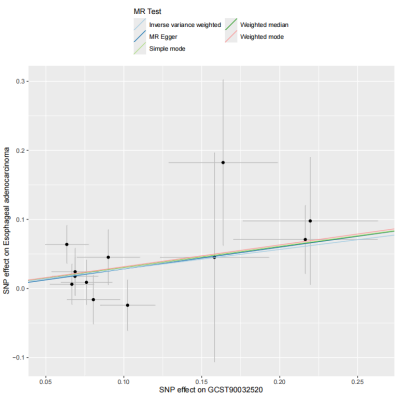

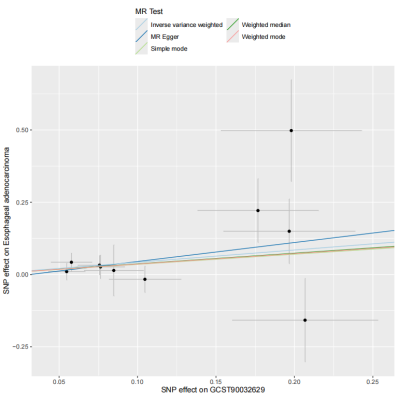

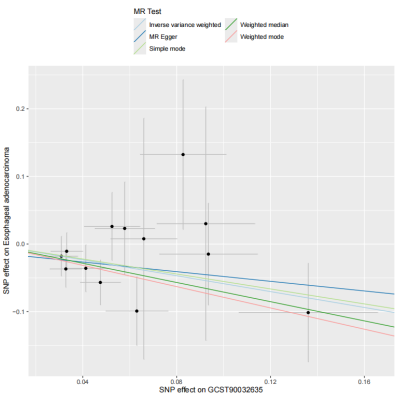

Supplement: Supplementary file 1 — Supporting Information 1. Figure S1: The scatterplots represent genetic IVs association between GM and EC/EAC. EC, esophageal cancer; EAC, esophageal adenocarcinoma. [file IJOG-2026-5967802-s004.docx]

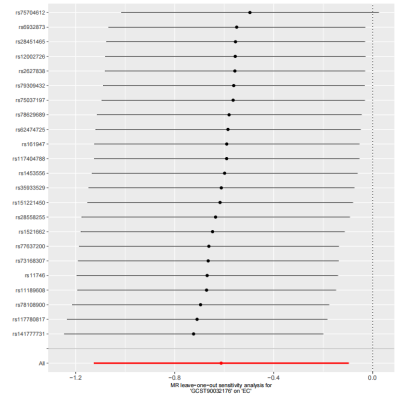

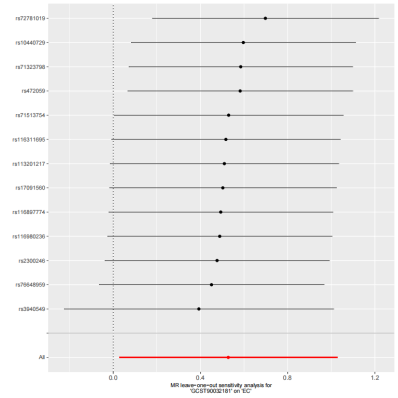

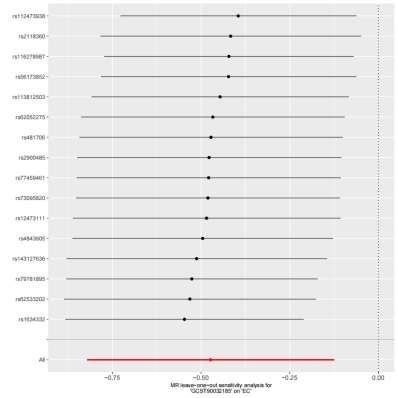

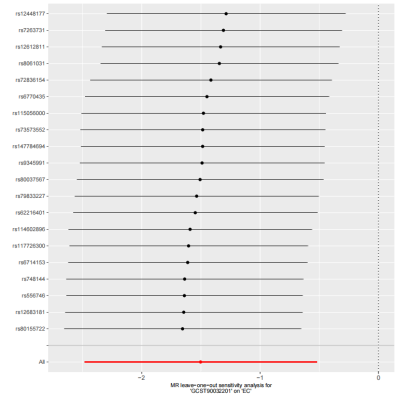

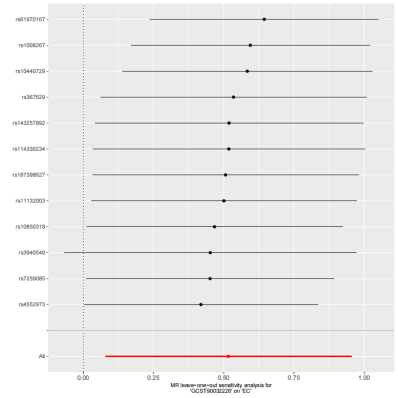

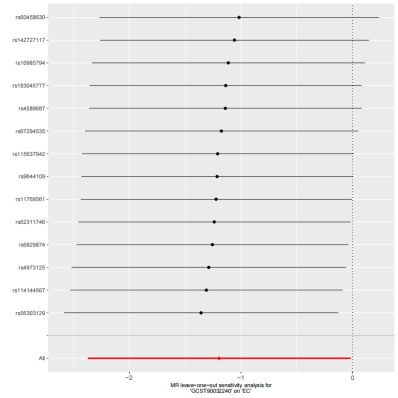

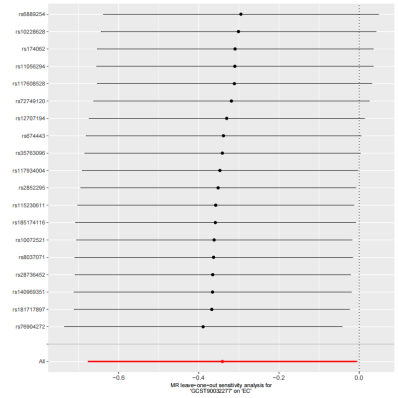

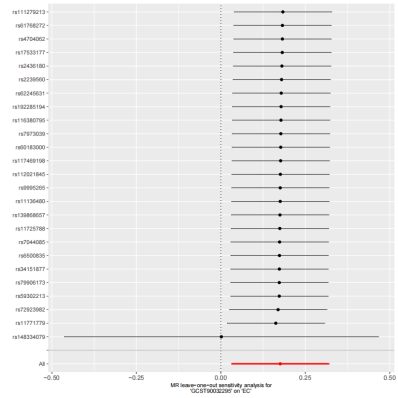

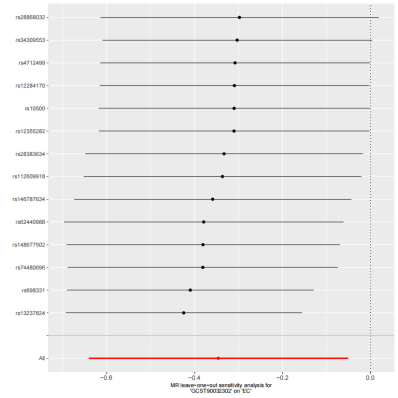

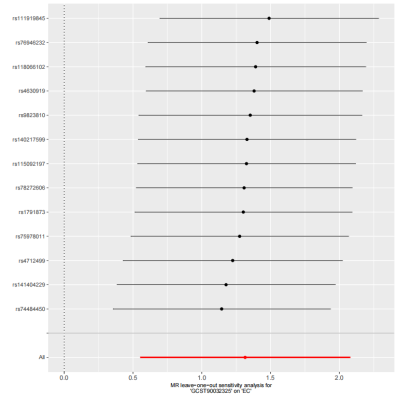

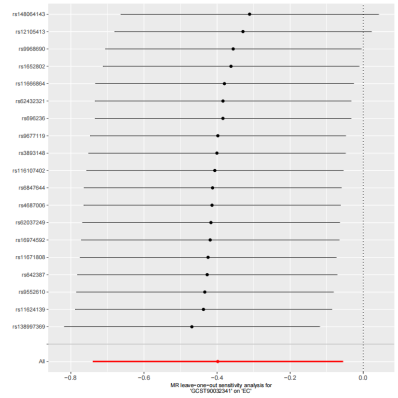

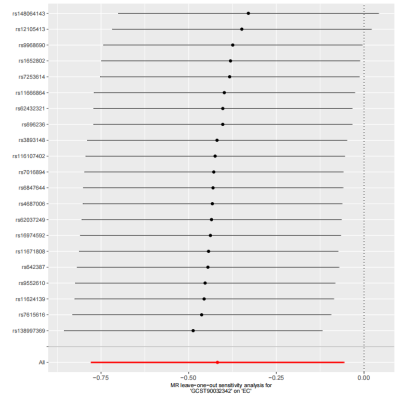

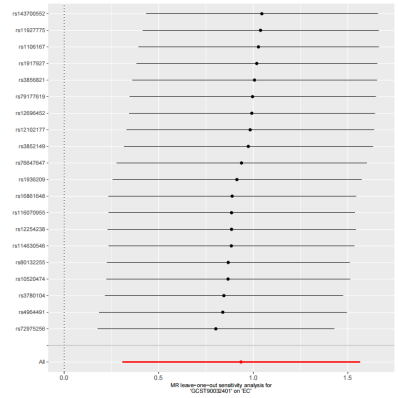

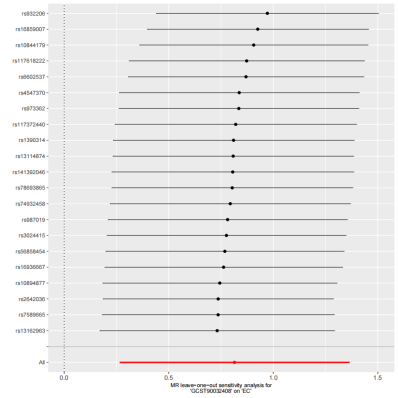

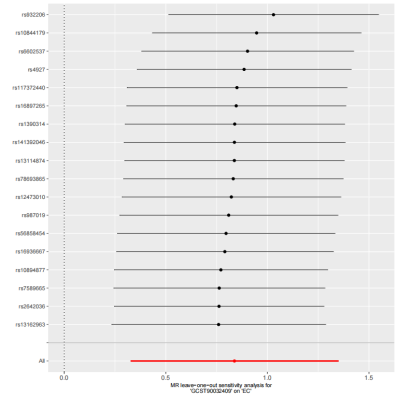

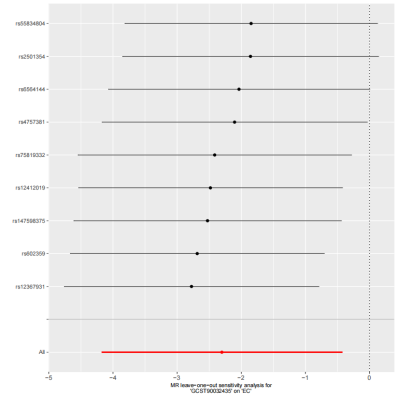

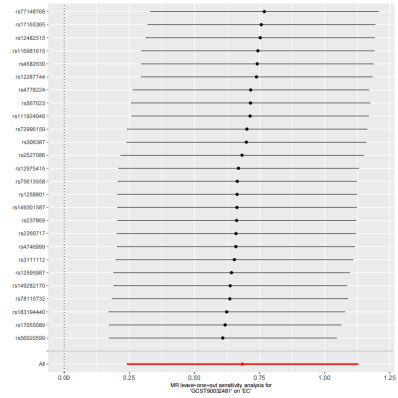

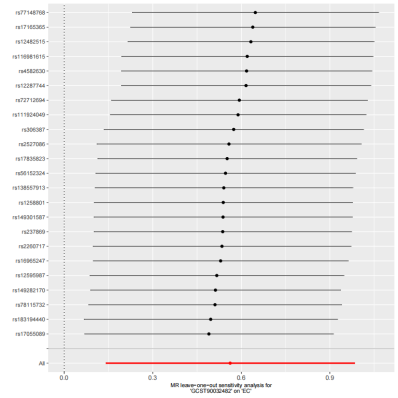

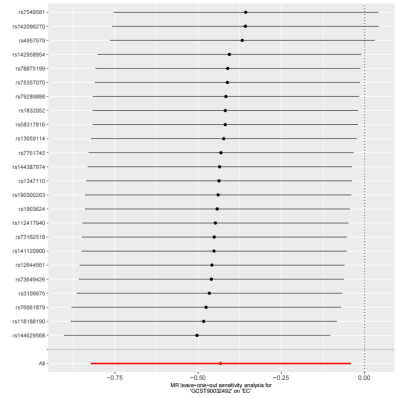

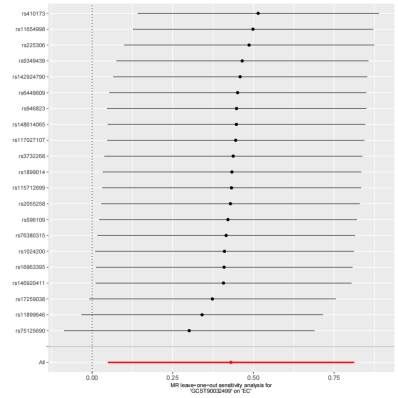

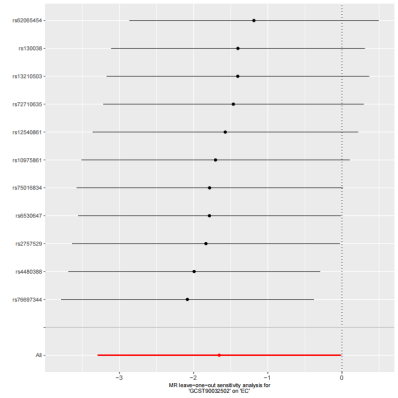

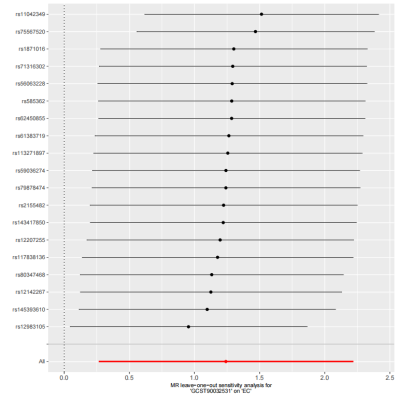

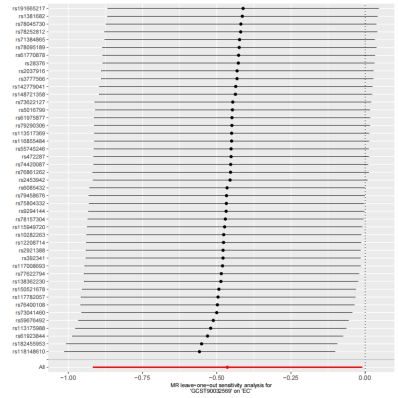

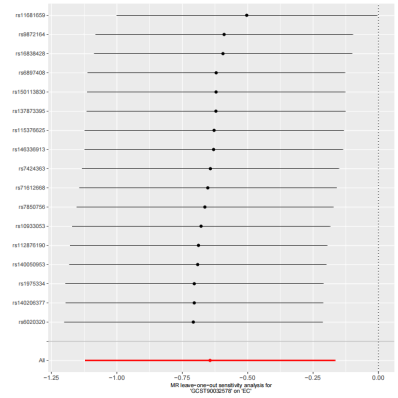

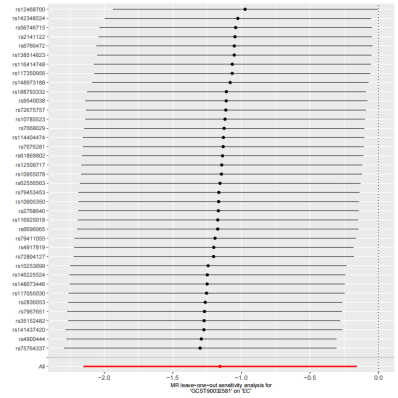

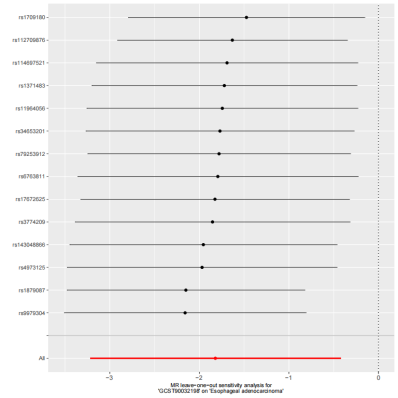

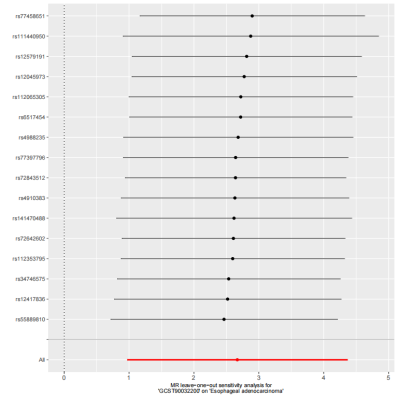

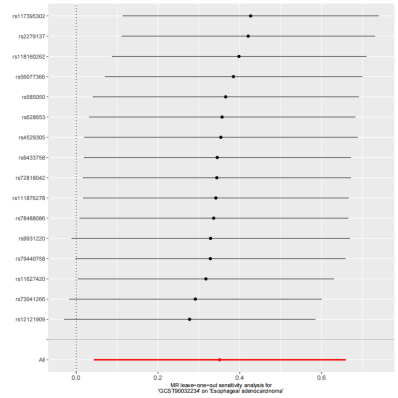

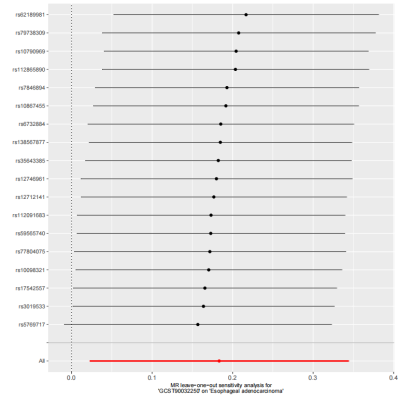

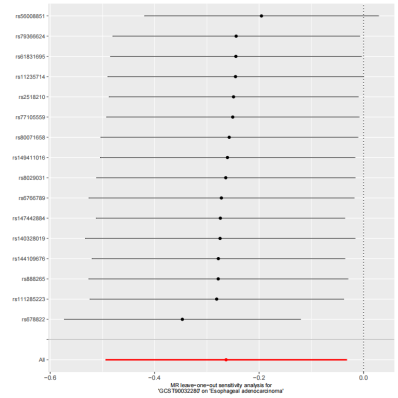

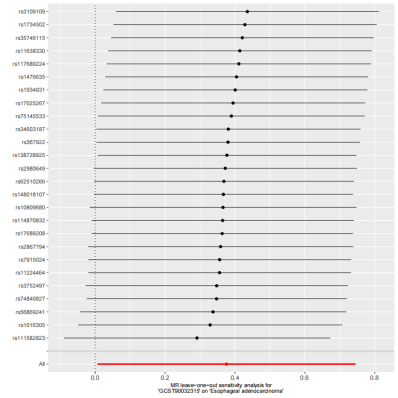

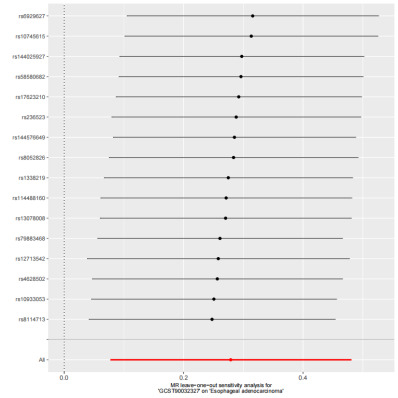

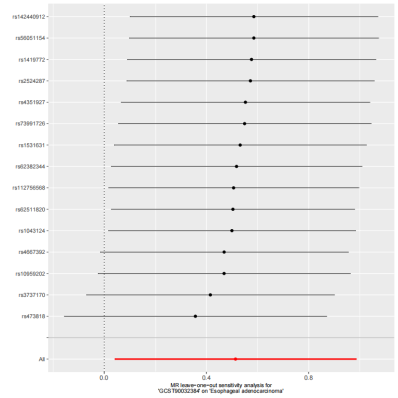

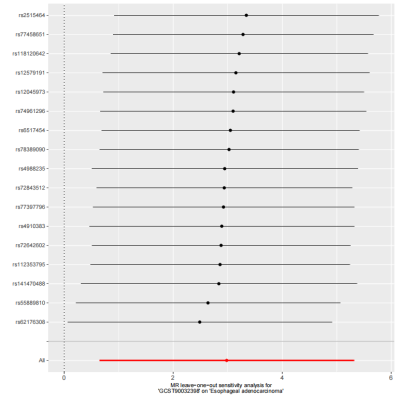

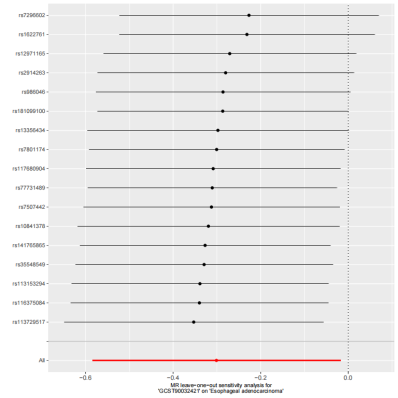

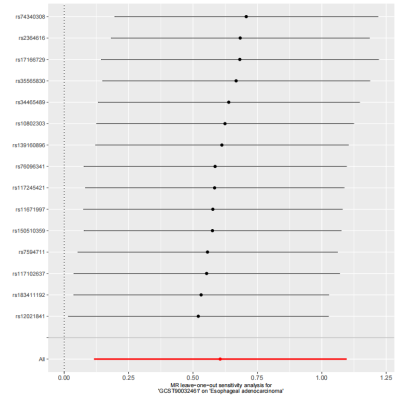

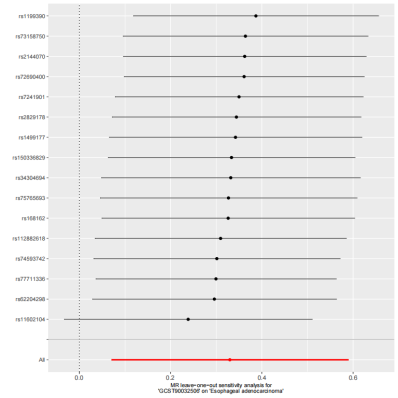

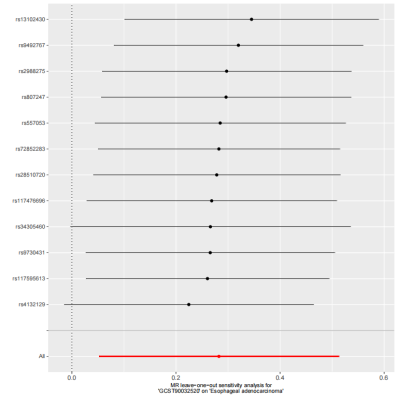

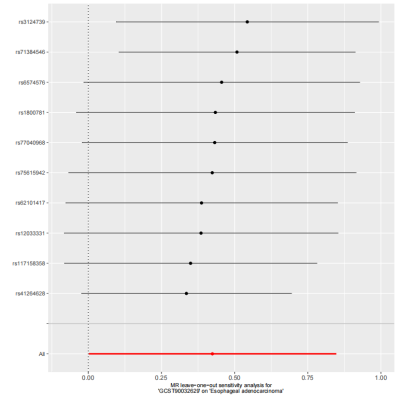

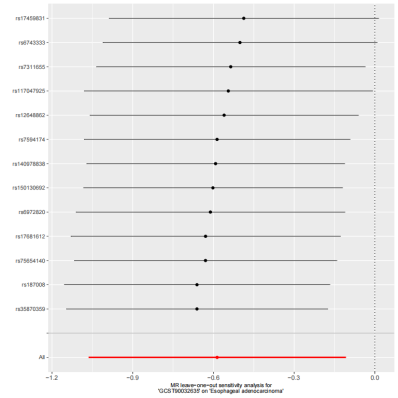

Supplement: Supplementary file 2 — Supporting Information 2. Figure S2: MR leave‐one‐out sensitivity analysis plots for GM on EA/EAC. EC, esophageal cancer; EAC, esophageal adenocarcinoma. [file IJOG-2026-5967802-s003.docx]

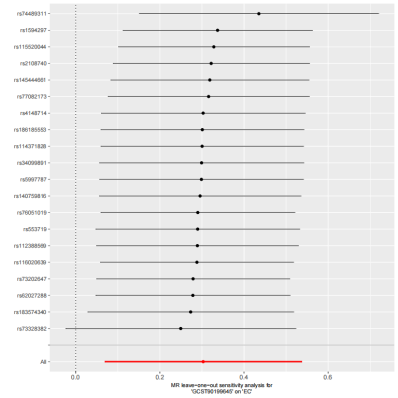

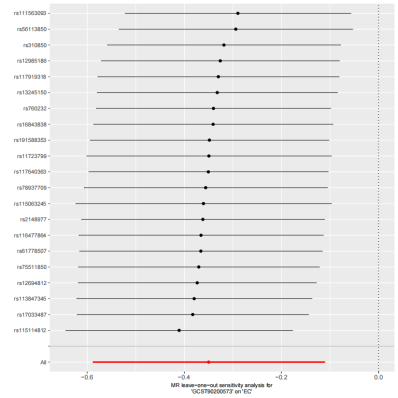

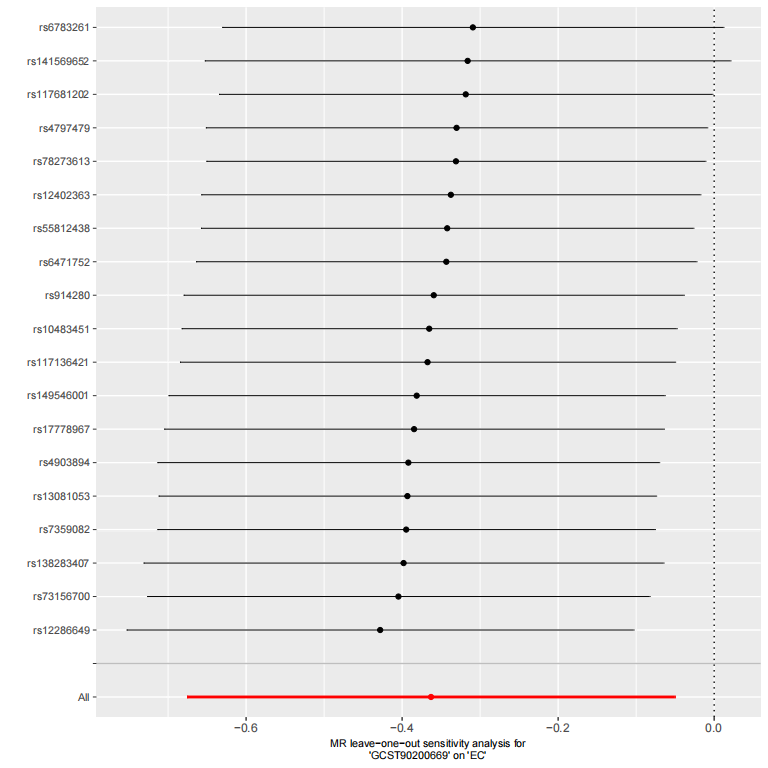

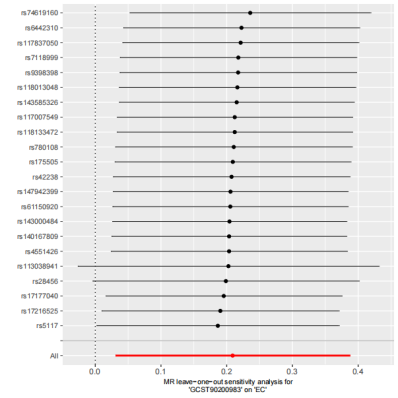

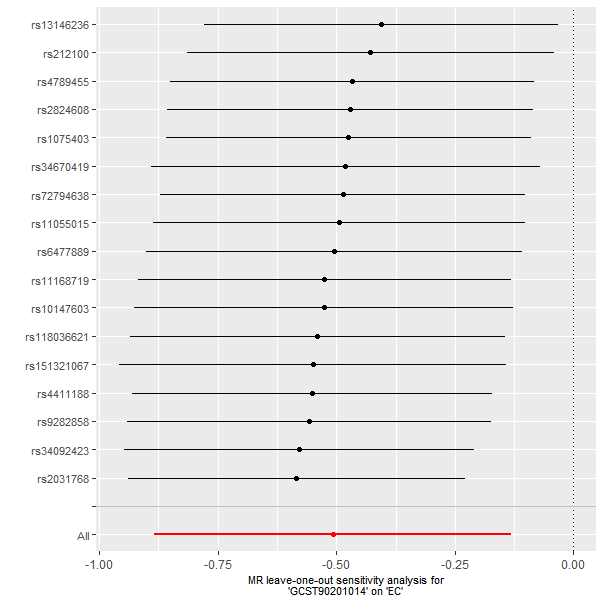

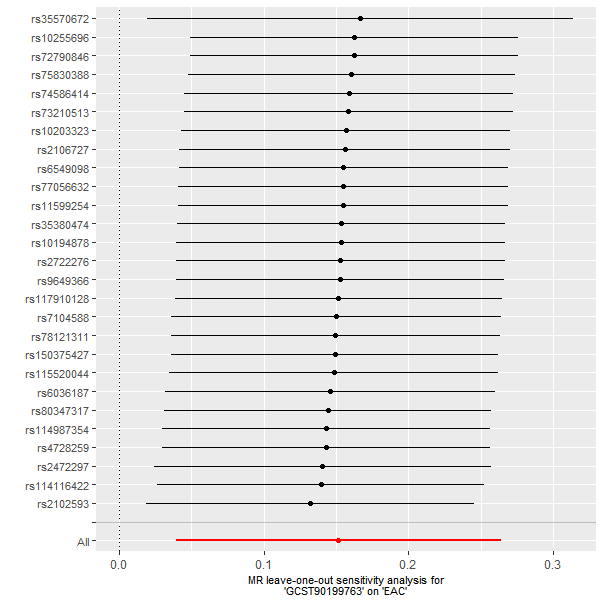

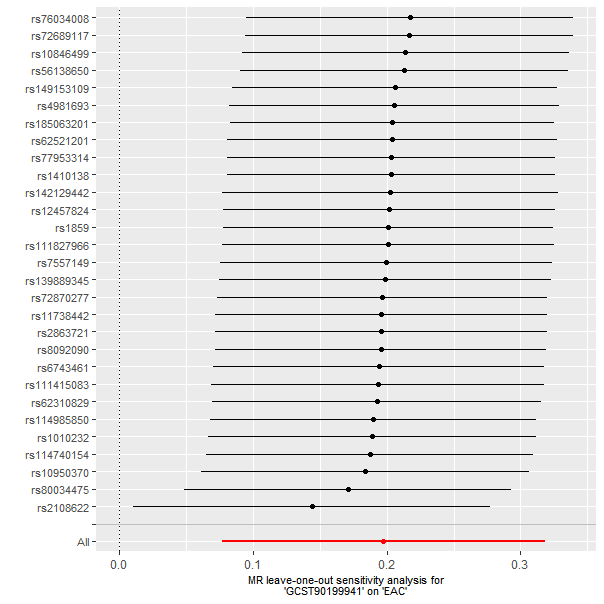

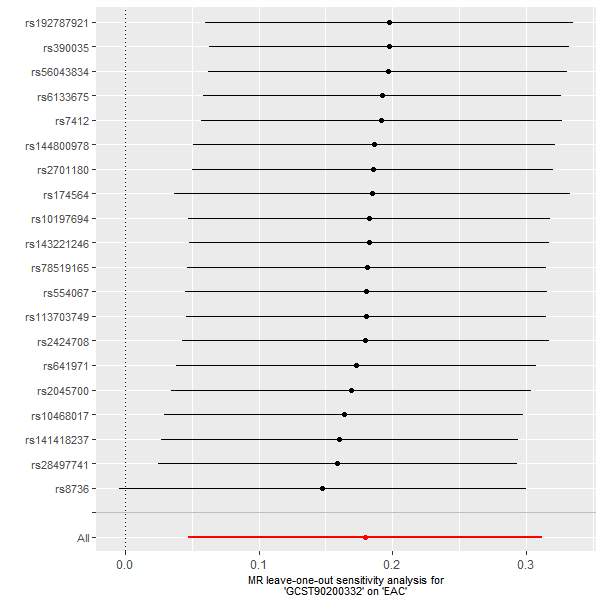

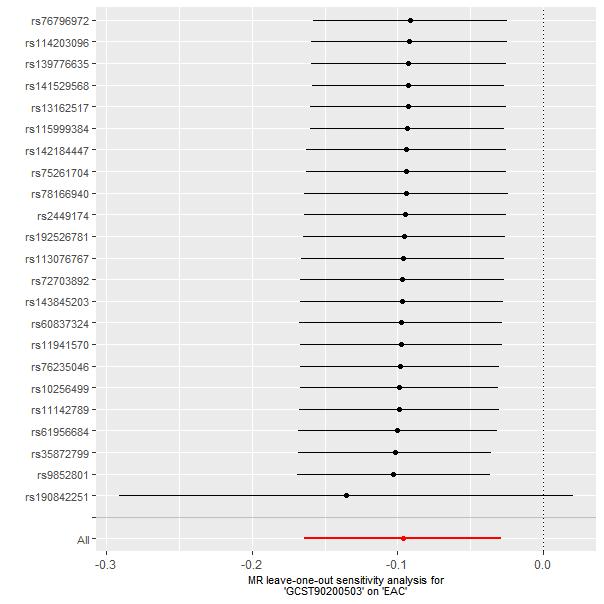

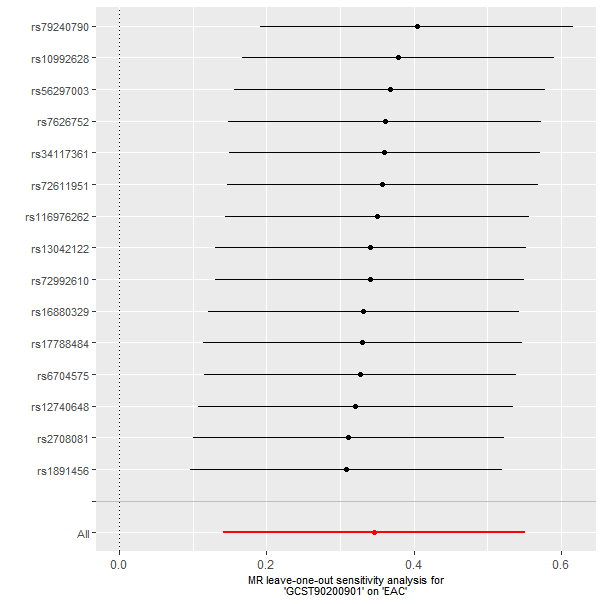

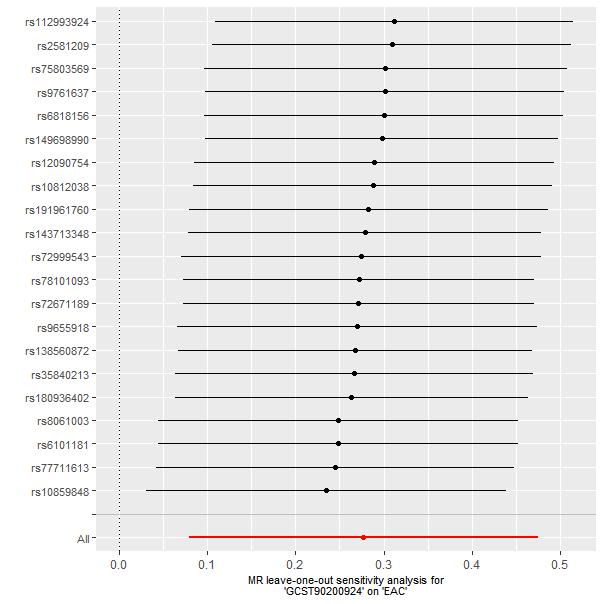

Supplement: Supplementary file 3 — Supporting Information 3. Figure S3: MR leave‐one‐out sensitivity analysis plots for metabolites on EA/EAC. EC, esophageal cancer; EAC, esophageal adenocarcinoma. [file IJOG-2026-5967802-s002.docx]
